# Supplementary material for: Recombinant Expression in Pichia pastoris System of Three Potent Kv1.3 Channel Blockers: Vm24, Anuroctoxin, and Ts6
Source: J Fungi (Basel). 2022 Nov 17;8(11):1215. doi: 10.3390/jof8111215 (PMC9697831; doi:10.3390/jof8111215)
Supplement: Supplementary file 1 [file jof-08-01215-s001.zip › jof-2000120-supplementary.pdf]

Journal of Fungi

Article

Recombinant expression in *Pichia pastoris* system of three potent Kv1.3 channel blockers: Vm24, Anuroctoxin, and Ts6.

Jesús Borrego[1], Muhammad Umair Naseem[1], Al Nasar Ahmed Sehgal[1], Panda Lipsa Rani[1], Kashmala Shakeel[1], Attila Gáspar[2], Cynthia Nagy[2], Zoltan Varga[1] and Gyorgy Panyi[1,*].

[1] Department of Biophysics and Cell Biology, Faculty of Medicine, University of Debrecen, Debrecen, Hungary.
[2] Department of Inorganic and Analytical Chemistry, Faculty of Science and Technology, Institute of Chemistry, University of Debrecen, Debrecen, Hungary.
* Correspondence: panyi@med.unideb.hu (G.P.); Tel.: (+36)(52) 258-603

Journal of *Fungi*

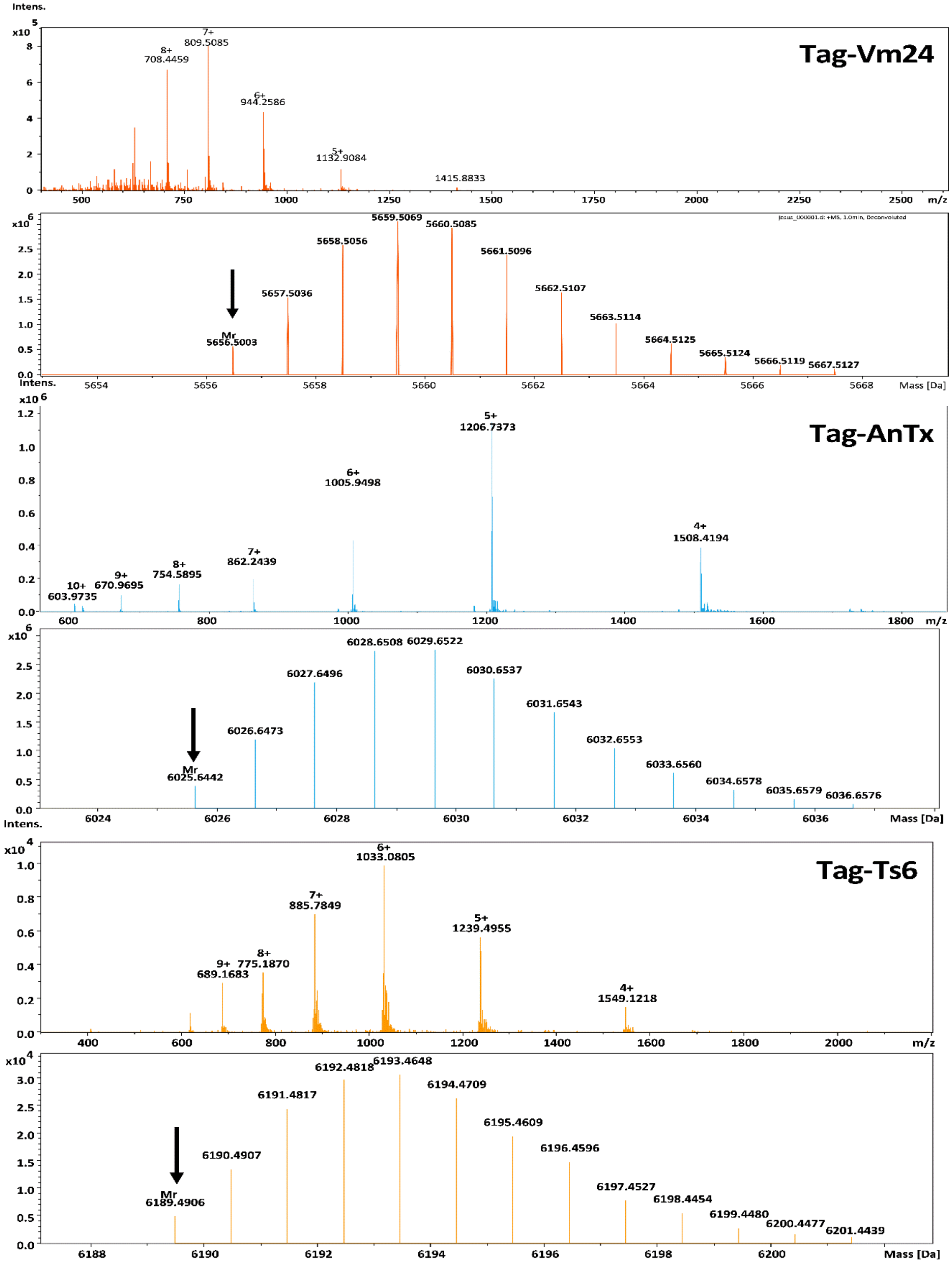

Figure S1. Mass spectrometry analysis of the tagged peptides. The raw (top) and deconvoluted (bottom) spectrums are shown for each peptide. Deconvoluted spectrum shows a reconstructed, neutral specrum. Black arrow points to the monoisotopic molecular mass.

Journal of Fungi

MDPI

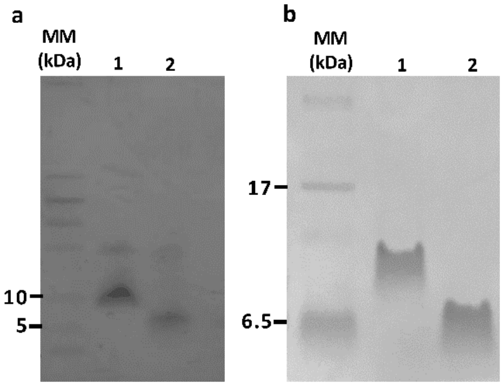

Figure S2. Tricine SDS-PAGE analysis of tagged and untagged Ts6 and AnTx. a) Recombiant Ts6. MM: Molecular marker (kDa), line 1: Tag-Ts6, and line 2: Untag-Ts6. b) Recombiant AnTx. MM: Molecular marker (kDa), line 1: Tag-AnTx, and line 2: Untag-AnTx.

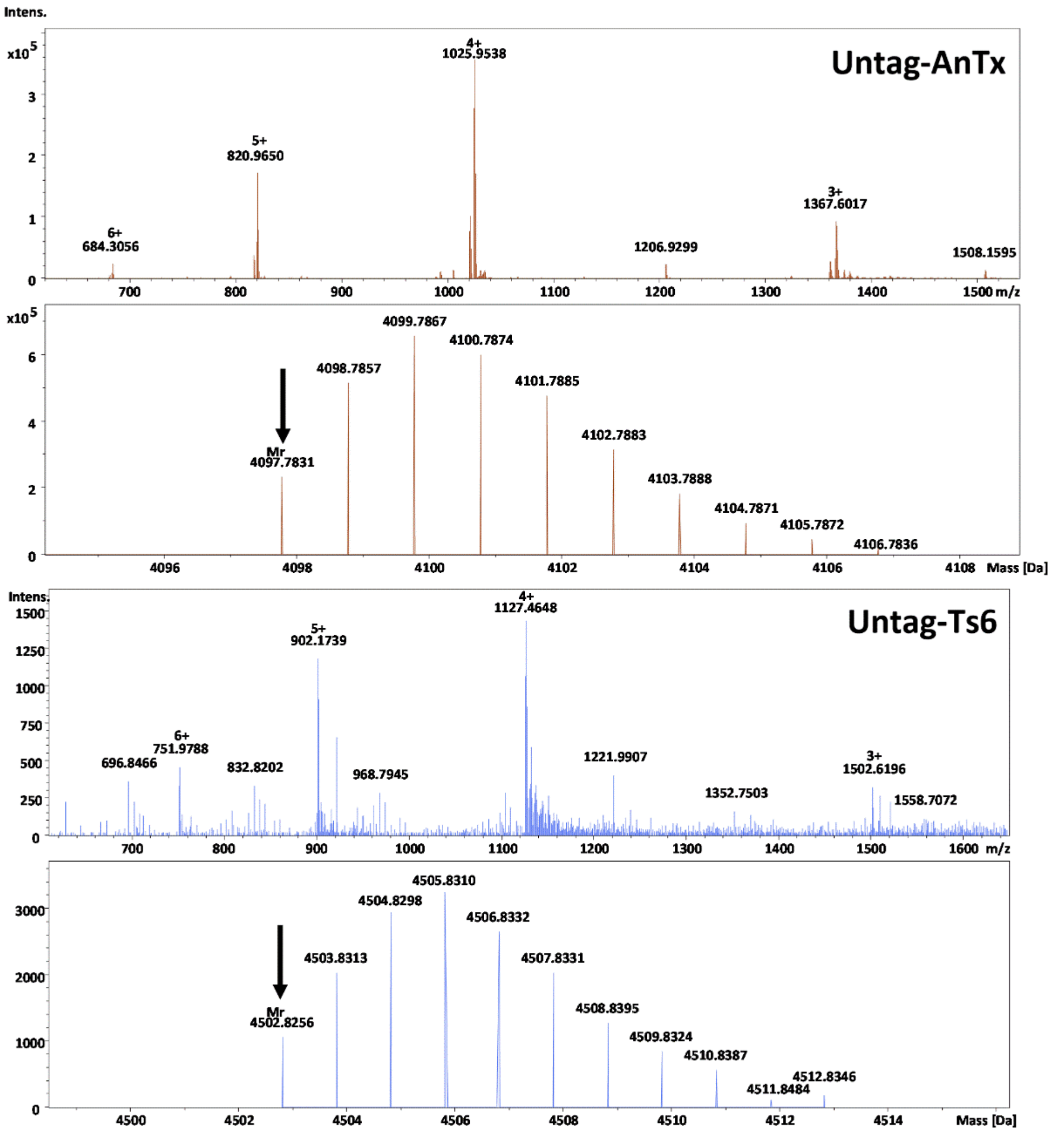

Figure S3. Mass spectrometry analysis of the untagged peptides. The raw (top) and deconvoluted (bottom) spectrums are shown for each peptide. Deconvoluted spectrum shows a reconstructed, neutral specrum. Black arrow points to the monoisotopic molecular mass.
